# Supplementary material for: Towards unified AI-driven fracture mechanics: the extended deep energy method (XDEM)
Source: Nat Commun. 2026 Aug 15;17:8492. doi: 10.1038/s41467-026-76748-1 (PMC13484471; doi:10.1038/s41467-026-76748-1)
Supplement: Supplementary file 3 [file 41467_2026_76748_MOESM3_ESM.zip › pic/Method/RK_architecture.pdf]

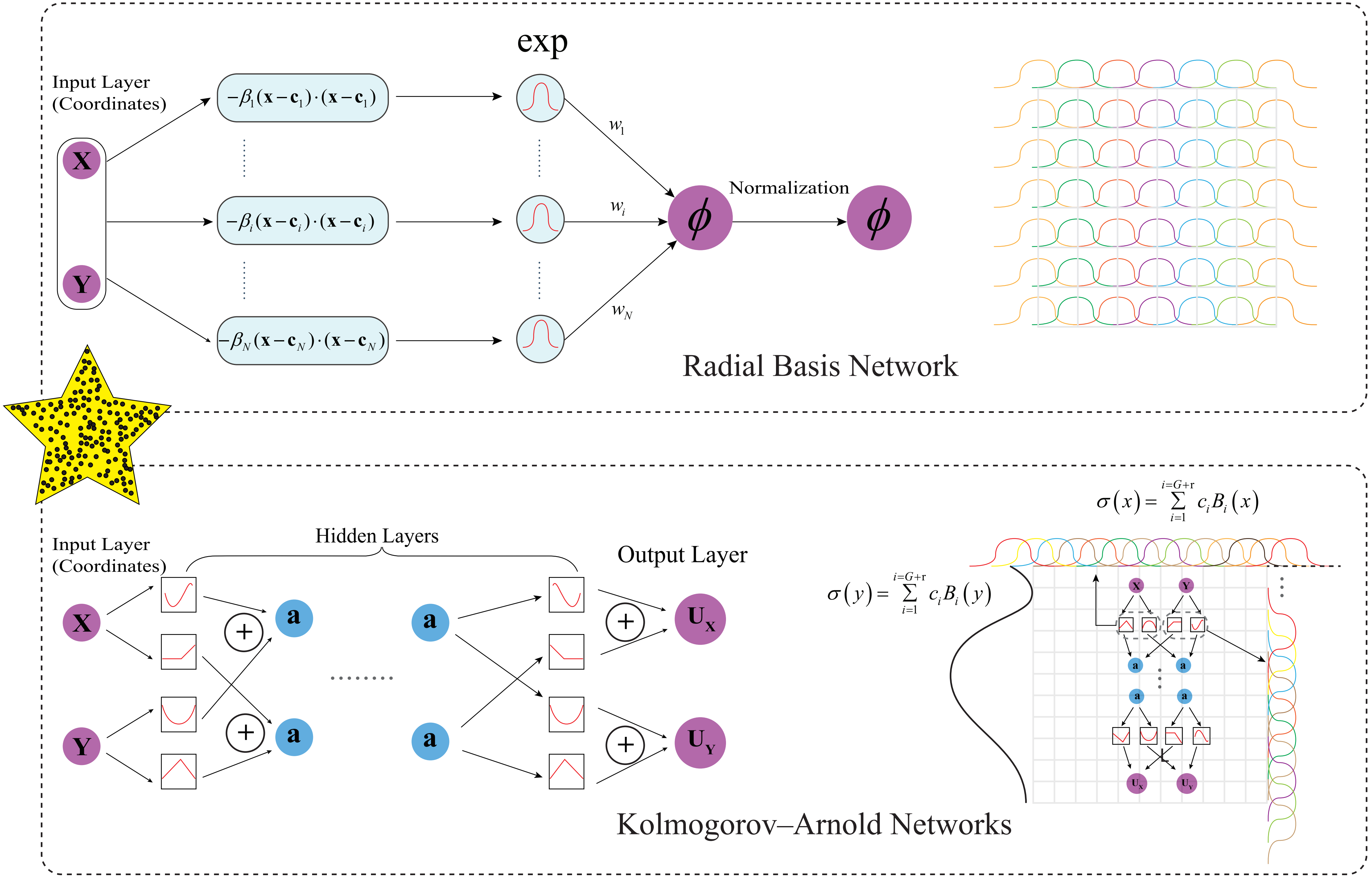

exp
Input Layer
(Coordinates)
X
Y
$-\beta_1(\mathbf{x}-\mathbf{c}_1)\cdot(\mathbf{x}-\mathbf{c}_1)$
$-\beta_i(\mathbf{x}-\mathbf{c}_i)\cdot(\mathbf{x}-\mathbf{c}_i)$
$-\beta_N(\mathbf{x}-\mathbf{c}_N)\cdot(\mathbf{x}-\mathbf{c}_N)$
w_1
w_i
w_N
ϕ
Normalization
ϕ
Radial Basis Network
Input Layer
(Coordinates)
Hidden Layers
Output Layer
X
Y
a
a
a
a
U_X
U_Y
$\sigma(x) = \sum_{i=1}^{i=G+r} c_i B_i(x)$
$\sigma(y) = \sum_{i=1}^{i=G+r} c_i B_i(y)$
Kolmogorov–Arnold Networks
